# Lead halide perovskite light-emitting diodes: navigating challenges and opportunities for next-generation display and lighting

**DOI:** 10.3389/fchem.2026.1952893

**Published:** 2026-09-04

**Authors:** Linxiang Yang, Xiuting Wu, Beichen Yuan, Tianjun Hu, Cunyun Xu, Qingsong Shan, Haibo Zeng

**Affiliations:** 1 School of Materials and Energy, Guang’an Institute of Technology, Guang’an, China; 2 MIIT Key Laboratory of Advanced Display Materials and Devices, Institute of Optoelectronics & Nanomaterials, School of Materials Science and Engineering, Nanjing University of Science and Technology, Nanjing, China

**Keywords:** device enhancement, display and lighting applications, lead halide perovskite, light-emitting diodes, synthesis approaches

## Abstract

Lead halide perovskite light-emitting diodes (PeLEDs) have emerged as highly promising candidates for next-generation displays and solid-state lighting, achieving record-high external quantum efficiencies (EQEs) exceeding 30%. The perovskites possess outstanding optoelectronic properties, such as high photoluminescence quantum yields (PLQY), narrow full-width at half maximum (FWHM), and efficient radiative recombination, driven fundamentally by the unique Pb^2+^ orbital configuration that imparts direct bandgaps and intrinsic defect tolerance. However, commercializing PeLEDs in next-generation displays and lighting is bottlenecked by dual-challenges in material and device: intrinsically, the soft lattice stemming from ionic soft lattice is prone to phase degradation, ion migration, and environment-induced dissociation, triggering severe non-radiative recombination, luminescence failure and environmental toxicity; extrinsically, unbalanced carrier injection, interfacial energy-level mismatch, and Joule heating effect jointly inflict low luminescence efficiency and rapid device decay. This review offers a systematic overview of multi-dimensional strategies developed to resolve this key contradiction. From a synthetic chemistry perspective, we summarize intrinsic lattice stabilization via compositional engineering, surficial ligand passivation and core-shell heterostructures, as well as solvent-free mechano-synthesis pathways. From a device engineering standpoint, we highlight charge-transport layer optimization, self-assembled monolayer (SAM) functionalization for interfacial-defect passivation and balanced carrier injection, alongside photonic waveguiding management. Finally, we discuss future perspectives on atomic-level mechanism characterization, ligand-mediated surface reconstruction, and closed-loop lifecycle management, offering a comprehensive roadmap toward the high-performance, long-term stability and scalable commercialization of next-generation PeLEDs for display and lighting technologies.

## 1 Introduction

**FIGURE 1:**
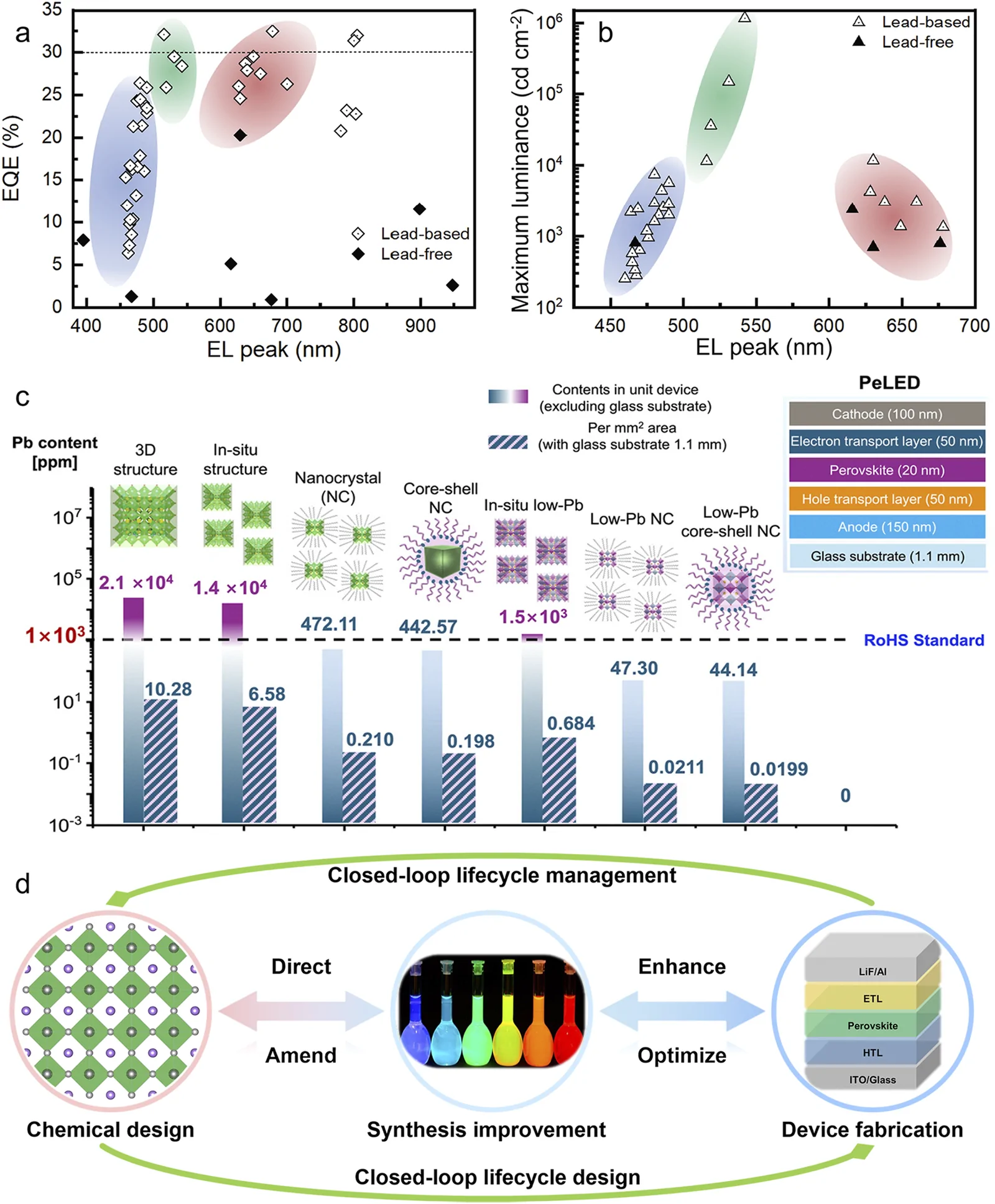
Performance disparity and quantitative lead content analysis across PeLED architectures. **(a,b)** The EQE and luminance statistics of PeLEDs (Liu et al., 2026). **(c)** Quantitative analysis of Pb content in PeLEDs compared to the RoHS standard (1000 ppm) (Shi et al., 2025). **(d)** The logical framework integrating chemical design, material synthesis, device engineering, and closed-loop lifecycle management.

Light-emitting diodes (LEDs) have revolutionized lighting and display technologies, among which the perovskite LEDs (PeLEDs) acquired increasing attention owing to the prominent advantages of wide color gamut (Wei et al., 2025), excellent flexibility, and high efficiency (Jang and Jang, 2023; Sun et al., 2023; Wang Y.-K. et al., 2024; Wei et al., 2025). Perovskite, the critical material of PeLED, intrinsically presents characteristics of tunable direct bandgap, high defect tolerance, superior photoluminescence quantum yield (PLQY), narrow full-width at half maximum (FWHM), and compatible low-cost solution processing (Zhang L. X. et al., 2023; Liu et al., 2025; Shi et al., 2025). Profiting from the concerted innovations in material synthesis and device physics, the PeLED performance has achieved rapid evolution in external quantum efficiency (EQE) and luminance (Gan et al., 2025; Gao et al., 2024; Jiang et al., 2025; Ke et al., 2025; Peng et al., 2026; Sun et al., 2025). The synergy between efficient radiative recombination via defect passivation and advanced light extraction strategies serves as the cornerstone for driving PeLEDs beyond their theoretical efficiency limits (Han et al., 2022; Li et al., 2024). Consequently, record-high EQEs of 42.9% (Peng et al., 2026) and 32.5% (Jiang et al., 2025) have been achieved for green and red devices respectively (Figures 1a,b; Liu et al., 2026), coupled with untra-high maximum luminance exceeding 10^6^ cd m^-2^, thereby paving the way for the further commercialization of perovskite display and lighting technologies.

However, translating these bench-scale efficiency triumphs into commercially viable displays and solid-state lighting, requires surmounting deep-seated stability and sustainability bottlenecks that originate from the intrinsic nature of perovskites. The dilemma lies characteristic of the ionic-crystal properties of perovskite and the toxicity of Pb element (Han et al., 2022; Maietta et al., 2025).

At the heart of this dilemma lies the dual nature of lead (Pb): On one hand, the unique electronic configuration of Pb establishes the electronic framework for efficient luminescence and defect tolerance (Fabini et al., 2020). On the other hand, the dynamic and relatively weak ionic bonding within the [PbX_6_]^4-^ inorganic framework imparts severe structural vulnerability, triggering defects, ion migration and sever phase instability. More critically, the spontaneous interface reactions between emissive material layer (EML) and electron-/hole-transport layer (ETL/HTL) accelerate the device degradation (Do et al., 2025; Yuan et al., 2025). Parallelly, the toxicological hazards and ecological risks of Pb leakage aggravate the environmental concerns (Woo et al., 2021), which have prompted stringent global regulatory standards, such as the European Union’s RoHS Directive (Directive 2011/65/EU) and China’s mandatory standard (GB 26572-2025), imposing a maximum lead limit of 0.1% by weight (1000 ppm) in homogeneous electronic products (Han et al., 2022; Zhang M. et al., 2025).

A quantitative evaluation of Pb concentration across representative PeLED stack architectures provides critical perspective on regulatory compliance. As presented in Figure 1c (Shi et al., 2025), an investigation of the relative Pb content across various perovskite compositions revealed that all nanocrystal LEDs fully complied with RoHS standards. This demonstrated that the contribution of Pb in ultrathin (20 nm) perovskite layer was negligible relative to the overall device architecture. Nevertheless, environmental safety thresholds for Pb leaching into aquatic and soil ecosystems are orders of magnitude more stringent under accidental damage, continuous operation, or post-consumer disposal. Consequently, given that securing Pb^2+^ lattice integrity and preventing leakage are the paramount requirement for commercialization, a multidimensional analysis encompassing chemical design, synthesis strategies, interfacial optimization, device fabrication, and closed-loop lifecycle management is essential to unlock the potential of next-generation PeLEDs, which precisely motivates this review.

This review provides a comprehensive overview integrating chemical design, material synthesis, device engineering, and closed-loop lifecycle management to address the critical challenges of PeLEDs (Figure 1d), with a particular emphasis on balancing application-oriented performance and sustainability. Specifically, the fundamental principles of Pb-management and photo-electronic characteristic improvement are first elucidated as pivotal design drivers. Next, we survey recent advances in material-level chemical design and eco-friendly synthesis, spotlighting strategies for lattice stabilization, ion-migration suppression, defect passivation and core-shell structure construction. We then examine device-level architecture engineering, with dedicated focus on charge-transport layer regulation, interfacial chemical stabilization, and photonic waveguiding management. Finally, a holistic perspective across the closed-loop lifecycle is comprehensively explored. This review aims to offer actionable insights to accelerate the commercial deployment of robust, eco-compatible PeLEDs in next-generation display and lighting technologies.

## 2 The mechanism of Pb in perovskite

**FIGURE 2:**
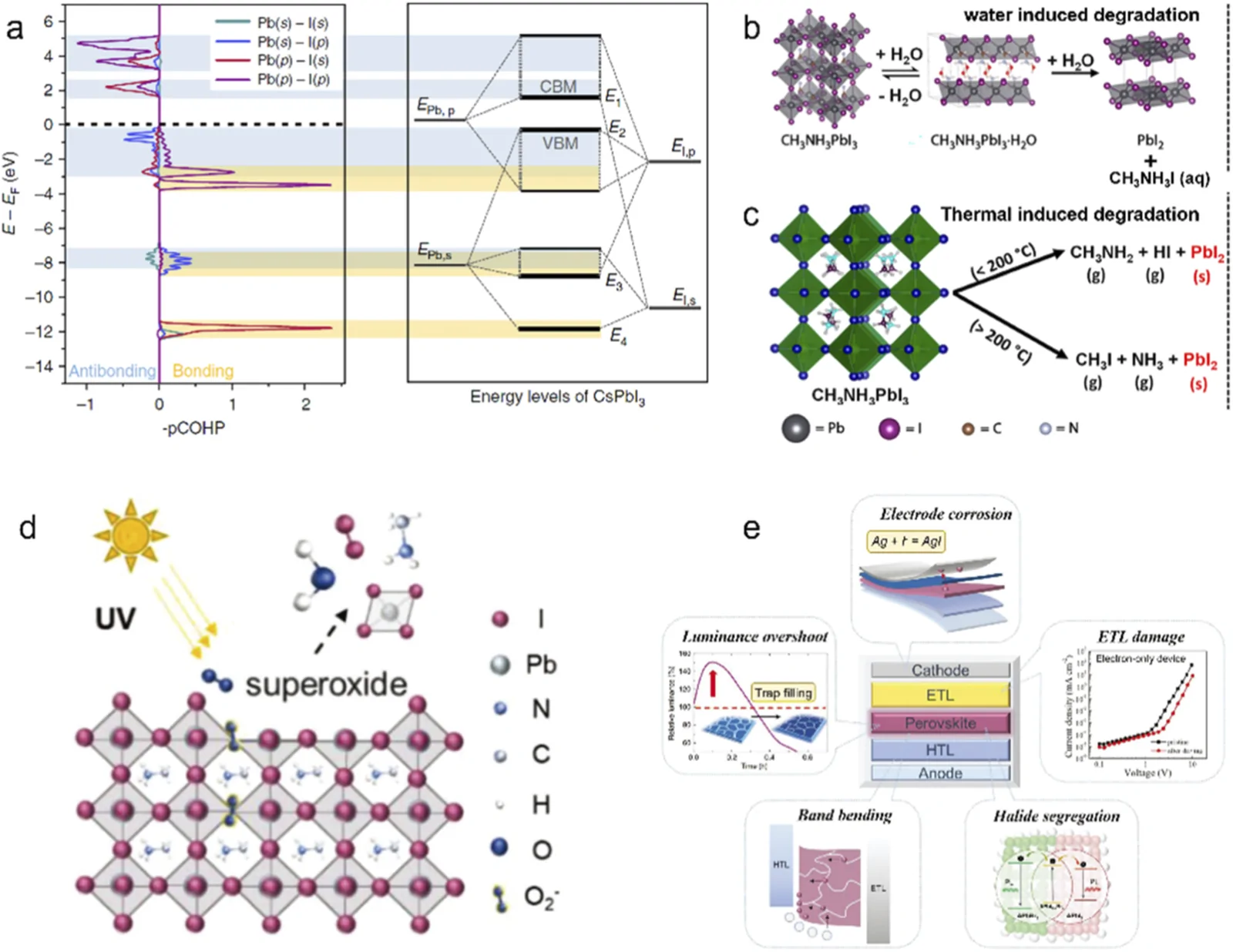
Electronic origins and multimodal degradation mechanisms of lead-halide perovskites. **(a)** The Orbital-resolved crystal orbital Hamiltonian population (Tao et al., 2019). **(b,c)** The water- and thermal-induced degradation mechanism of MAPbI_3_ (Ravi et al., 2020). **(d)** Schematic representations of perovskite crystal degradation under the effect of UV light (Kong et al., 2022). **(e)** Consequences of the electric-field induced ion migration for PeLEDs, including luminance overshoot, band bending, halide segregation, ETL damage, and electrode corrosion (Kong et al., 2022).

The remarkable electroluminescence efficiency exhibited by PeLEDs is rooted in the unique orbital configuration and electronic structure of Pb (Stranks and Snaith, 2015; Tao et al., 2019). As schematically illustrated in Figure 2a (Fabini et al., 2020), the valence band maximum is characterized by the strong antibonding hybridization of Pb 6s and I p orbitals, whereas the conduction band minimum is predominantly constituted by delocalized Pb 6p states (Umebayashi et al., 2003). This distinctive structure not only yields a direct bandgap optimized for efficient radiative transition but also imparts an intrinsic “defect tolerance” to the perovskite lattice, where dominant intrinsic defects create only shallow levels (Brandt et al., 2015; Yin et al., 2014).

Paradoxically, the unique ionic characteristic of perovskite, the lead-halide octahedron inorganic framework ([PbX_6_]^4-^), also serves as the intrinsic thermodynamic origin of their structural instability, wherein the highly dynamic and relatively weak coordination bonds inherently predispose the soft lattice to facile cation dissociation and severe halide migration (Li et al., 2022; Thiesbrummel et al., 2026). These defects not only act as potent non-radiative recombination centers that quench luminescence but also catalyze the irreversible decomposition of surrounding perovskite matrices (Mosquera-Lois et al., 2025). Significantly reducing the photoelectronic performance and device stability (Thiesbrummel et al., 2026). Consequently, the operational failure of PeLEDs is driven by the synergistic interplay of electrical, thermal, optical, and moisture-induced degradation pathways. Firstly, in the presence of moisture and oxygen, H_2_O easily penetrates the dynamic crystal lattice to form unstable hydrated phases, initiating irreversible decomposition into solid PbX_2_ and volatile amines (Figure 2b; Ravi et al., 2020). Concurrently, excited electrons reduce diffused O_2_ into reactive superoxide radicals (O^2-^), which deprotonate organic cations (MA^+^, FA^+^), trigger 3D perovskite lattice collapse and severely drop the PLQY (Aristidou et al., 2015; Kong et al., 2022). Secondly, the thermal effect during annealing or operating is proven to be the main factor causing perovskite decomposition, the volatilized organic cations promote the process of thermodynamically driven phase transition in high-performance perovskites (Figure 2c; Kundu and Kelly, 2020; Ravi et al., 2020), transitioning highly emissive black α−FAPbI_3_ phase to the opto-electronically inactive yellow δ-phase or lead-rich secondary phases CsPb_2_X_5_ (Li et al., 2026; Wang Z. et al., 2025). Joule heating generated under operational bias exacerbates this thermal degradation process (Woo et al., 2021). Thirdly, electro-photo processes severely compounded the degradation of emissive materials, functional layers and device structure. Non-equilibrium carriers generated under illumination, which drastically lower the thermodynamic barrier of ion migration, causing the diffusion of halide vacancy and decomposition of [PbX_6_]^4-^ framework (Figure 2d; Kong et al., 2022; Zheng and Kim, 2022). More seriously, operating bias facilitates the rapid, directional drift of reactive halides (I^−^, Br^−^, or Cl^−^) and the vacancy accumulation at the heterojunction interfaces, which induces the formation of deep-level defect states that severely quench radiative-recombination, and simultaneously phase segregation that detrimentally shift spectra and reduce stability of devices (Liang et al., 2025). Furthermore, sustained, high-density charge injection triggers the electrochemical reduction of bonding Pb^2+^ into dissociative Pb^0^ (Mirabelli et al., 2025). These electrochemically generated Pb^0^ aggregates not only function as deleterious non-radiative recombination centers that quench electroluminescence, but also act as autocatalytic sites that accelerate the irreversible decomposition of the adjacent perovskite matrix or destructive reactions of transport layer/interface, thereby severely compromising the EQE (Figure 2e; Kong et al., 2022; Yuan et al., 2022). Ultimately, these synergistic, self-accelerating degradation pathways highlight the paramount importance of developing active chemical synthesizing strategies, advanced device fabricating, and robust encapsulation to ensure the safe and sustainable commercial deployment of PeLED technologies.

## 3 Enhancement strategies for high-performance materials

To overcome the challenges of ion migration, phase decomposition and performance degradation, synthetic chemistry has emerged as the most critical tool to secure the structural integrity of Pb^2+^ and prevent device failure. This section systematically reviews state-of-the-art synthetic methodologies, ranging from lattice doping and surface ligand engineering to green, solvent-free processing, with a primary emphasis on the underlying chemical mechanisms that lock the lead-halide framework.

### 3.1 Intrinsic lattice optimization

**FIGURE 3:**
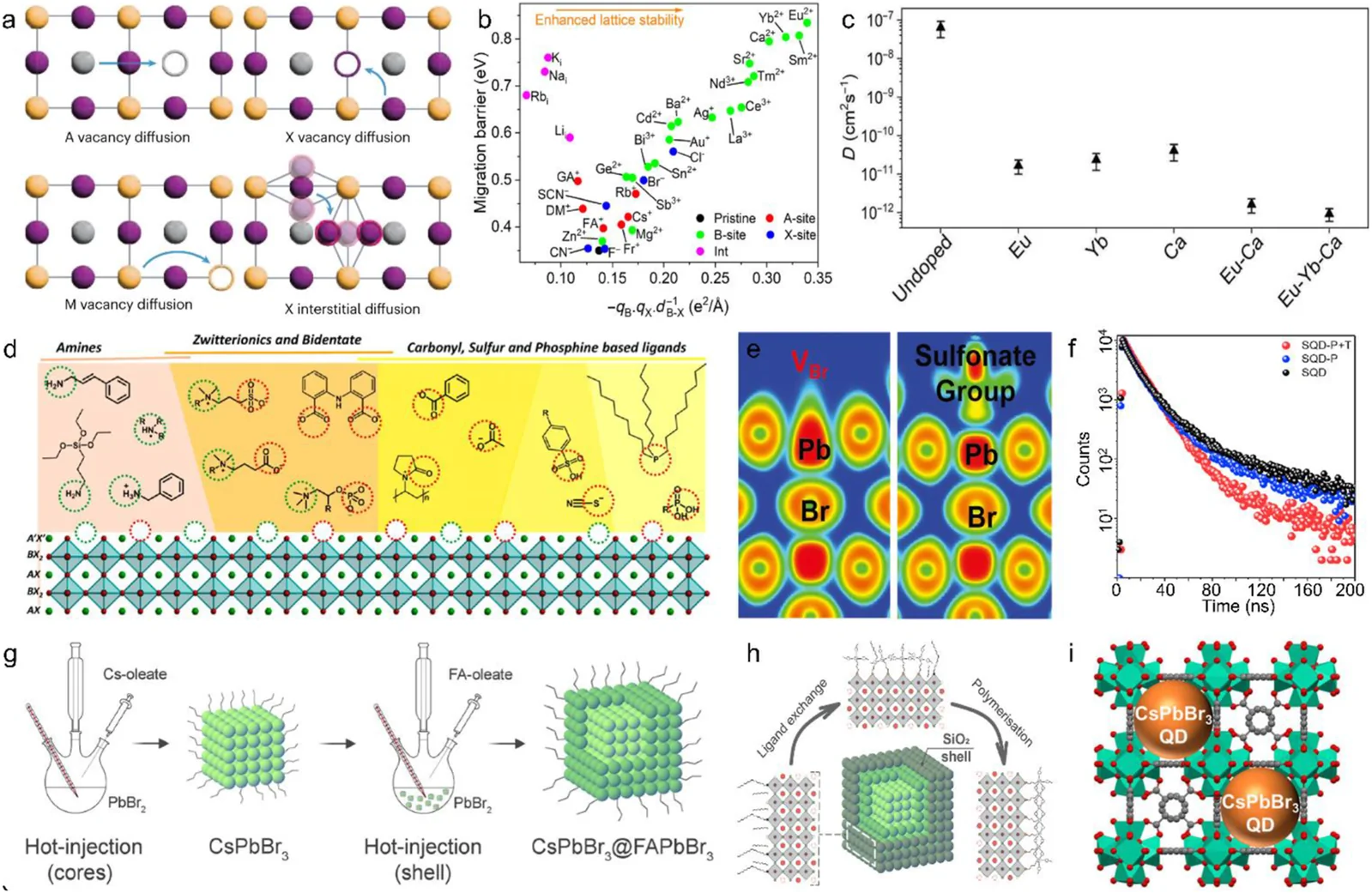
The synthesis enhancement strategies of perovskite. **(a)** The mechanisms of lattice-defect types and ion transport in perovskite (Thiesbrummel et al., 2026). **(b)** Comparison of I^−^ migration barriers in MAPbI_3_ perovskite with various ion dopants (Liang et al., 2025). **(c)** Ion diffusivity in pristine, rare-earth-doped, and multi-element-doped perovskites derived from simulations (Liang et al., 2025). **(d)** The summary of ligands employed in surface passivation; the functional groups of ligands are classified according to their binding sites (Fiuza-Maneiro et al., 2023). **(e)** The DFT-calculated electron cloud schematic diagram of perovskite quantum dots (QDs) (Yang et al., 2019). **(f)** Schematic illustration of the time-resolved transient PL spectra of perovskite QDs after ligand passivation (Xie et al., 2022). **(g,h)** Schematic illustrations of the synthesis and the silica shelling process of the CsPbBr_3_@FAPbBr_3_ core-shell nanocrystals (NCs) (Tatarinov et al., 2025; Wu R. et al., 2026). **(i)** Schematic representation of perovskite in metal-organic frameworks (MOFs) (CsPbBr_3_@UiO-66) (Lin et al., 2025).

The intrinsic thermodynamic vulnerability of the [PbX_6_]^4-^ inorganic framework represents the primary structural catalyst for defect generation, phase transitions and electric-bias-driven ion migration. To surmount these multidimensional degradation pathways, material stabilization strategies, predominantly compositional engineering and B-site ion doping, must be fundamentally understood through their atomic- and electronic-level regulation of lattice distortion, defect formation, and ion migration. As demonstrated by the ion-transport mechanism in Figure 3a (Thiesbrummel et al., 2026), the various lattice defects, including vacancies and interstitials, serves as the primary conduits for photo- or electric-induced ionic redistribution or phase separation.

B-site cation engineering acts as an intrinsic chemical lever to simultaneously elevate the kinetic ion-migration barrier and minimize the defect density. Incorporating smaller homovalent or heterovalent transition metal cations (e.g., Mn^2+^, Zn^2+^, Sr^2+^, Nd^3+^, et al.) to partially replace Pb^2+^ contracted the unit cell (Futscher et al., 2020; Kovalenko et al., 2025; Li et al., 2020; Shen et al., 2019; Yang et al., 2021), effectively adjusting Pb-X bond lengths (Wang Y.-K. et al., 2024; Figure 3b), which concurrently raised the thermodynamic formation energy of native vacancies and creates a more tortuous diffusion pathway, thereby increasing the stability of lattice and suppressing the subsequent ion drift. Zheng et al. systematically unraveled the crucial physical mechanism (Liang et al., 2025), the B-site substitution, particularly with low-electronegativity lanthanide and alkaline-earth elements, notably strengthens local ionic interactions and restrains octahedral rocking motions, resulting in a 90% increase in the migration barrier of I^−^. Especially, the machine learning molecular dynamics revealed that the multiple-doped rare-earth system (Eu-Yb-Ca) reduced the interstitial iodine diffusivity by five orders of magnitude (Figure 3c), comparable to the values observed in lower-dimensional perovskites, but without sacrificing the three-dimensional charge transport properties of the host lattice (Hu et al., 2025).

Complementary to B-site modification, A-site compositional alloying governs macroscopic crystallographic stability by modulating the structural tolerance factor and octahedral connectivity (Thiesbrummel et al., 2026). Incorporating smaller inorganic cations (e.g., Cs^+^ or Rb^+^) systematically decreased the Goldschmidt tolerance factor to the ideal cubic range, suppressing the thermal tilting of the [PbX_6_]^4-^ octahedra (Wang Z. et al., 2025), which successfully prevented the non-radiative conversion of black-phase α-FAPbI_3_ into the yellow non-radiative phase δ-FAPbI_3_ (Woo et al., 2021). Simultaneously, large organic spacer cations (PEA^+^, BA^+^) constructed dimensionally modulated quasi-2D Ruddlesden-Popper or Dion-Jacobson phase heterostructures (Tsai et al., 2016), which physically interrupted the three-dimensional ion migration pathways, thereby suppressing electric-field-driven halide phase segregation and non-radiative recombination decay (Karlsson et al., 2021).

Component engineering and cation doping strategy reinforces the perovskite matrix by distorting perovskite lattice and increasing the migration barrier, simultaneously suppressing the defect generation and improving the photoelectronic properties of perovskite.

### 3.2 Surface stabilization

The high specific surface area in NCs renders their surfaces highly susceptible to coordination state disintegration (Chu et al., 2025). The resulting undercoordinated surface halogen vacancies serve as non-radiative trap centers and promoted the escape and dissociation of Pb, severely quenching photo- and electro-luminescence of devices (Li et al., 2026; Zheng and Kim, 2022). Synthesis strategies encompassing ligand engineering, core-shell construction, and crystalline matrix encapsulation were implemented to form robust protective chemical and physical barriers (Wu X. T. et al., 2026; Zhang X. et al., 2024; Zhang X. et al., 2025).

The coordinate ligands to a certain extend dictate the colloidal crystallization kinetics: ligands with optimal binding affinities dynamically regulate monomer supersaturation, directing anisotropic crystal facet growth, and suppressing defective sub-phases (Song et al., 2018). Figure 3d illustrated the classification of ligands based on their specific surface-binding sites (Fiuza-Maneiro et al., 2023), in which ammonium ligands anchor to A-cation sites, whereas carbonyl-, sulfur-, phosphine-based ligands coordinate to undercoordinated X-vacancies. The multi-dentate, zwitterionic, or short-chain aromatic surfactants are also proved as the advanced ligands in passivating undercoordinated surface states (Kim et al., 2022; Li et al., 2017; Song et al., 2018; Yuan et al., 2019). In contrast to the weakly coordinated, highly dynamic oleic acid (OA) and oleylamine (OAm) pairs that suffer from spontaneous desorption, Zeng et al. proposed a pioneering “Br-equivalent” ligand strategy to synthesize low defect CsPbBr_3_ QDs with utilizing dodecylbenzene sulfonic acid. Theoretical calculations (Figure 3e) and experimental characterization demonstrated that the strongly ionic sulfonate heads interacted vigorously with lead ions, which successfully excluded the electron cloud and alleviated the defect of the electronic structure, thereby the final QDs maintaining a high PLQY >90% without obvious deterioration (Yang et al., 2019). Furthermore, the sharply increased surface energy in strong confinement QDs created a high-density V_X_ and V_Pb_, which readily triggered thermodynamic phase transitions and electric-field-induced ion migration. Tian et al. developed a dual-ligand synergistic post-treatment strategy to improve the stability of QDs and reduce the electric field-induced Stark effect of the device (Xie et al., 2022). The PPA^+^ and TBA^+^ strongly bonded to Pb^2+^ and I^−^, systematically reconstructing the surface of QDs and passivating the V_Pb_ and V_I_. The rigid aromatic rings constructed a robust steric barrier that effectively suppresses detrimental ion migration, hence synergistically regularized the exciton binding energy to mitigate Auger recombination (Figure 3f), ultimately delivering spectrally stable, high-efficiency pure-red PeLEDs.

Core-shell hetero-structural construction and host-matrix encapsulation offer higher-dimensional structural paradigms for defect deactivation, dielectric confinement, and environmental isolation. Specifically, the epitaxial growth of structurally compatible perovskite derivatives (such as Cs_4_PbBr_6_ or FAPbBr_3_) established an exceptional lattice registry with the CsPbBr_3_ core, effectively passivating undercoordinated surface traps while mitigating non-radiative recombination (Ahmed et al., 2021; Zhang et al., 2020). Aleksandr et al. synthesized FAPbBr_3_@FAPbBr_3_ core-shell NCs (Figure 3g) to strengthen the stability (Tatarinov et al., 2025). By virtue of near-zero lattice mismatch, the epitaxial shell effectively passivated surface defect traps and reinforced structural phase integrity without introducing foreign chemical impurities. The robustness against moisture, thermal, and photolytic stresses of NCs could be further enhanced by coating with amorphous oxide shells (e.g., SiO_2_, TiO_2_) or hydrophobic polymer (e.g., PMMA, polystyrene), expanding the applications in flexible display (Cai et al., 2025; Patel et al., 2023; Shan et al., 2020). As demonstrated in Figure 3h, the Pb-S bonding between perovskite and MPTMS elevated the vacancy formation energies, which in turn improved the overall stability of QDs.

Complementing these strategies, MOFs provided a tailor-made, periodically porous host matrix for perovskite QDs. The nanometer-sized MOF pores imposed strict physical confinement that regulates QD crystallization dynamics and precludes agglomeration. Concurrently, the coordinate bonding from organic linkers within the MOF pore walls passivated undercoordinated surface sites, hence being proved as another effective strategy in regulating crystallization dynamics, passivating surface trap states, and physically blocking environmental moisture and oxygen for perovskite QDs (Figure 3i; Chen et al., 2022; Lin et al., 2025). This spatial confinement strategy not only preserved the intense green photoluminescence of the CsPbBr_3_ QDs for over 30 months under ambient conditions, but also successfully unlocked strong exciton-photon coupling, underscoring the massive potential of perovskite@MOF heterostructures in highly stable, next-generation display and quantum technologies (Lin et al., 2025).

Collectively, this integrated surface-engineering framework bridges precision ligand coordination, epitaxial shell passivation, and metal-organic framework to harmoniously eliminate non-radiative surface traps, suppress field-induced ion migration and Auger losses, and shield against environmental degradation, thereby establishing a robust foundation for high-performance and spectrally stable PeLEDs.

### 3.3 Sustainable synthesis and lead-free perovskite pursuing

**FIGURE 4:**
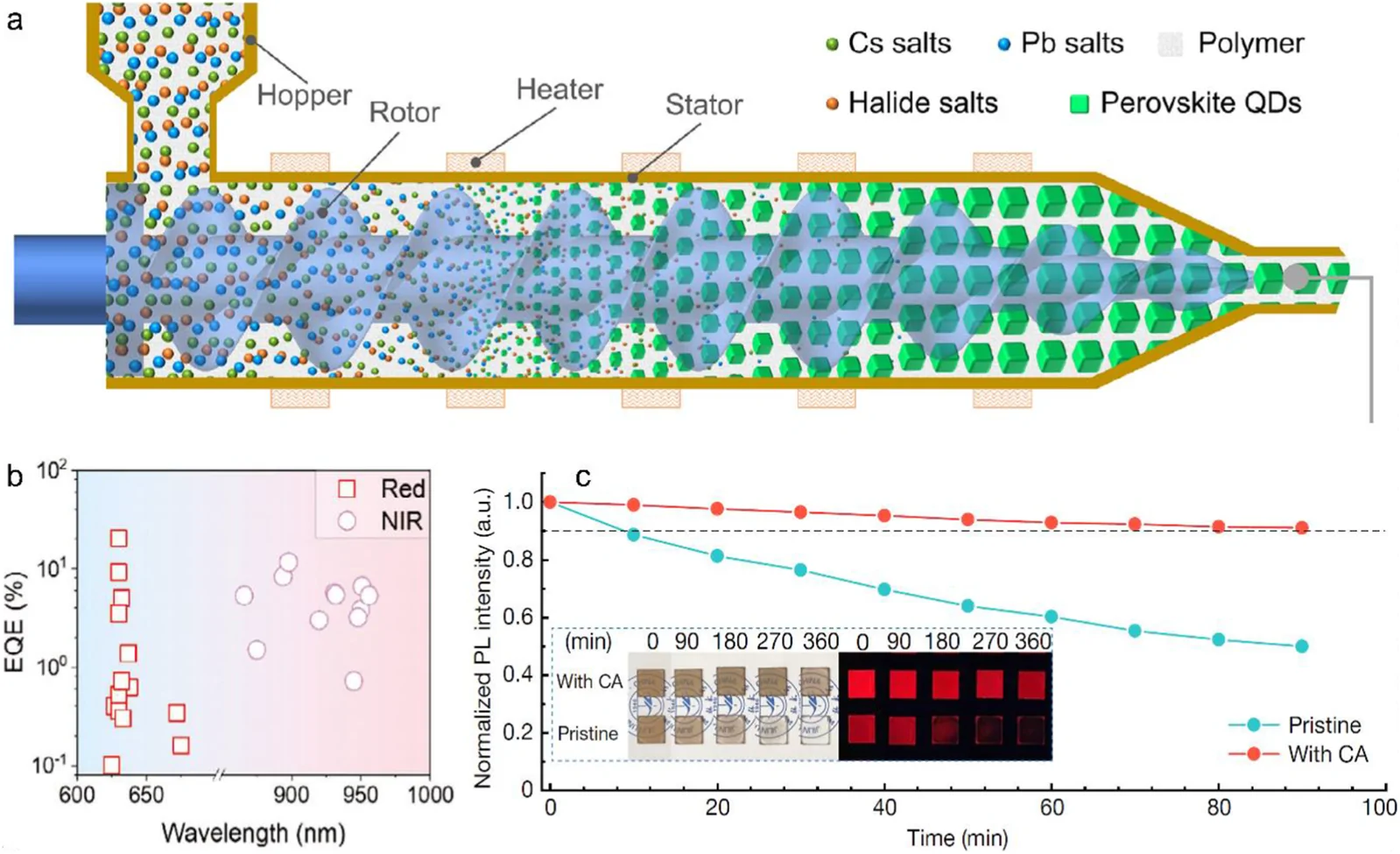
**(a)** Schematic illustration of the synthesis of perovskite QD/polymer composites using a screw extruder setup (Fan et al., 2025b). **(b)** EQEs of Sn-based PeLEDs (Tang et al., 2025). **(c)** Time-dependent normalized PL intensity measurements for the pristine and the TEA2SnI4 film for 90 min (Han et al., 2023).

Conventional solution-processed synthetic routes heavily rely on hazardous polar organic solvents, such as dimethylformamide and dimethyl sulfoxide, OA, OAm, generating volatile organic compounds and toxic liquid effluents, whose environmental and health concerns that often surpass those associated with the lead constituent itself (Bai et al., 2016; Sun et al., 2016). The low thermodynamic barrier and soft lattice of halide perovskites enable ambient mechanical energy to readily surmount the precursor activation barrier, driving solid-state interdiffusion and rapid crystallization. This advantage facilitates green, solvent-free mechanochemical synthesis, offering a low-carbon, eco-friendly pathway to high-performance perovskites. Early explorations demonstrated the wet-milling for synthesizing APbBr_3_ (A = Cs, FA) NCs, successfully bypassing the high-temperature requirements and solvent-heavy purification steps of traditional colloidal methods (Protesescu et al., 2018). Subsequently innovations evolved this approach into high-energy mechanochemical milling, yielding NCs with pronounced quantum-confinement effects and robust stability (Goyal et al., 2022). Song et al. synthesized CsPbBr_3_/ZrO_2_ hybrid phosphors via a rapid, solvent-free method under ambient conditions, wherein the robust and chemically inert ZrO_2_ matrix effectively preserved the initial perovskite lattice, achieving enhanced PL properties alongside improved thermal stability. Furthermore, they proposed a solvent-free “raw material selection-synthesis design-product process” strategy, realizing the scalable, single-step fabrication of uniform, low-defect, and highly luminescent QD/polymer nanocomposites with industrial manufacturing viability (Fan et al., 2025b). As illustrated in Figure 4a, continuous mechanochemical processing harnesses mechanical shear stress to induce *in situ* chemical metathesis between precursor salts and subsequent regulated crystallization, ultimately yielding monodisperse perovskite QDs uniformly embedded within the host polymer matrix (Fan et al., 2025b). Complementing this continuous process, they introduced a surface-medium anchoring strategy that delivered a twofold functional advantage (Fan et al., 2025a): the high organophilic compatibility of functionalized organosilicon microspheres with polymers facilitated the formation of uniform, crack-free composite films; and the high density of surface anchoring sites on these microspheres ensures spatial isolation of the *in situ* formed perovskite QDs, effectively suppressing aggregation-induced emission quenching and ion migration.

In addition, achieving holistic environmental sustainability in PeLEDs fundamentally demands transitioning from toxic lead frameworks toward low-lead and lead-free perovskite light-emitters (Zhang et al., 2026). In particular, Sn-based PeLEDs have achieved milestone EQEs surpassing 20% in the red and near-infrared windows (Figure 4b; Tang et al., 2025). By leveraging tautomeric-mixture-coordination-induced electron localization strategy, the Sn-based PeLEDs obtained a record EQE of 20.29%, approaching the efficiencies of Pb-based ones (Han et al., 2023). The strengthened electronic effects and mitigated non-radiative recombination originated from the robust chemical coordination between cyanuric acid and Sn^2+^ on the perovskite surface, which facilitated the high-efficiency and low-defect density structure. As presented in Figure 4c (Han et al., 2023), the treated film retained its 90% initial PL intensity within 90 min, indicating outstanding stability in operating PeLEDs, and providing insights into the stabilization of Sn-perovskites.

In summary, targeted synthetic strategies spanning lattice locking and interfacial encapsulation are essential to reinforce the lead-halide framework, thereby suppressing both bias-driven ion migration and undercoordinated Pb dissociation. Synergistically coupling atomic-level doping with robust multi-site ligand coordination effectively eliminates vacancy traps and constructs dense chemical barriers against hazardous Pb leakage. Ultimately, coupling advanced interfacial passivation paradigms with green, solvent-free mechanosynthesis, while actively pursuing non-toxic perovskites, paves a viable, sustainable pathway toward next-generation PeLEDs.

## 4 Strategies for long-term device stability

The synthetic chemistry strategies establish the intrinsic PLQY and thermodynamic stability of perovskite, the systematic device optimization is still required to meet the high-performance PeLEDs. The device degradation originated from the coupling of following disadvantages: electro-activated ion migration, interfacial-structure degradation, trap-induced nonradiative recombination, and unmitigated Joule heating, which could be settled via exciton-dynamics matching, carrier-transport-layer engineering, interfacial optimization and device-structure improvement (Li et al., 2026; Woo et al., 2021; Zheng and Kim, 2022).

**FIGURE 5:**
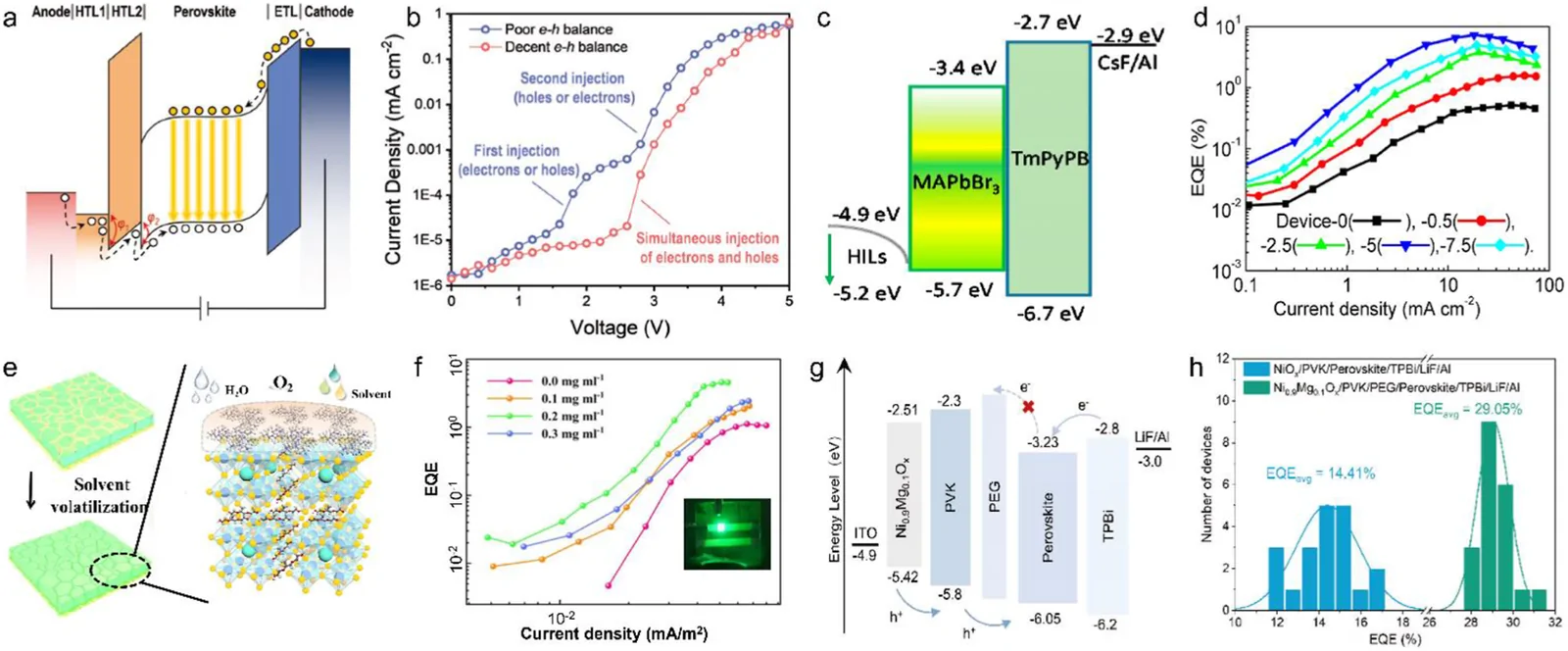
The device improvement approaches for PeLEDs. **(a,b)** An energy diagram of a working PeLED under perfect e-h balance, and the I-V curves of PeLEDs with poor and decent e-h balances (Chen et al., 2021). **(c,d)** The energy level diagram and the property of EQE-J curves of the PeLED based on MAPbBr_3_ (Peng et al., 2017). **(e)** Schematic diagram of the CsPbBr3 crystal structure after modification (Guo et al., 2025). **(f)** The EQE-J curves for PeLEDs with varying concentrations of the polyethylenimine (PEI) interlayer (Guo et al., 2025). **(g)** Schematic diagram of Ni_0.9_Mg_0.1_O_x_ transport layer and polyethylene glycol (PEG) in regulating energy-level and eliminating carrier leakage (Bai et al., 2023). **(h)** The EQE histogram of twenty devices (Bai et al., 2023).

In operating devices, injected carriers dynamically activated intrinsic lattice defects in perovskite and trigger uncontrolled ion migration, representing a primary origin of field-induced spectral drift in PeLEDs under electrical bias (Birkhold et al., 2018; Vashishtha and Halpert, 2017). Moreover, electrochemically generated defects (V_X_, Pb^0^) constituted deep non-radiative trap states that quenched radiative excitons and resulted in pronounced efficiency roll-off (Dong et al., 2020). Figure 5a exhibited the scheme of perfect e-h radiative recombination, where the electrons and holes reached the EML concurrently and radiatively recombine, without any carrier loss at the interface (Chen et al., 2021). The current density-voltage (J-V) inflection characteristics serve as a critical diagnostic tool for identifying carrier imbalances, where dual-step current jumps attributed to electron and hole injection into the perovskite layer, respectively (the blue curve in Figure 5b). Deploying precise interfacial engineering and energy-level matching could effectively minimize interfacial charge accumulation and suppresses trap-assisted non-radiative losses, ultimately appearing as a single jump in current density (the red curve in Figure 5b) (Chen et al., 2021).

### 4.1 Interfacial engineering for passivating interfacial defects and balancing carrier injection

Through the meticulous engineering of charge-transport layers (HTL/ETL), balanced carrier injection can be successfully realized, effectively suppressing both phase segregation and ion migration driven by EML charge accumulation (Li et al., 2024; You et al., 2026). Yang et al. reported a modified PEDOT:PSS as HTL, which increased the work function to 5.2 eV and promoted hole injection, leading to a 15-fold enhancement in PL intensity and 20-fold in EQE (Figures 5c,d; Peng et al., 2017). In a similar vein in Figures 5e,f, the PEI interlayer serves as an effective barrier shielding the active perovskite film against ambient moisture, oxygen ingress, and solvent-induced degradation (Guo et al., 2025). This device structure drove substantial improvements in overall optoelectronic performance, concurrently enhancing operational stability, EQE, luminescence, and the turn-on voltage. Furthermore, Xie et al. fabricated the Ni_0.9_Mg_0.1_O_x_/PVK gradient HTL structure (Figure 5g), which achieved significant improvement in EL performance (Bai et al., 2023). The adopted PEG eliminated carrier leakage and durably passivated the interfacial defects of perovskite, as exhibited in Figure 5h, the optimized PeLED consequently acquired a maximum EQE exceeding 30%.

**FIGURE 6:**
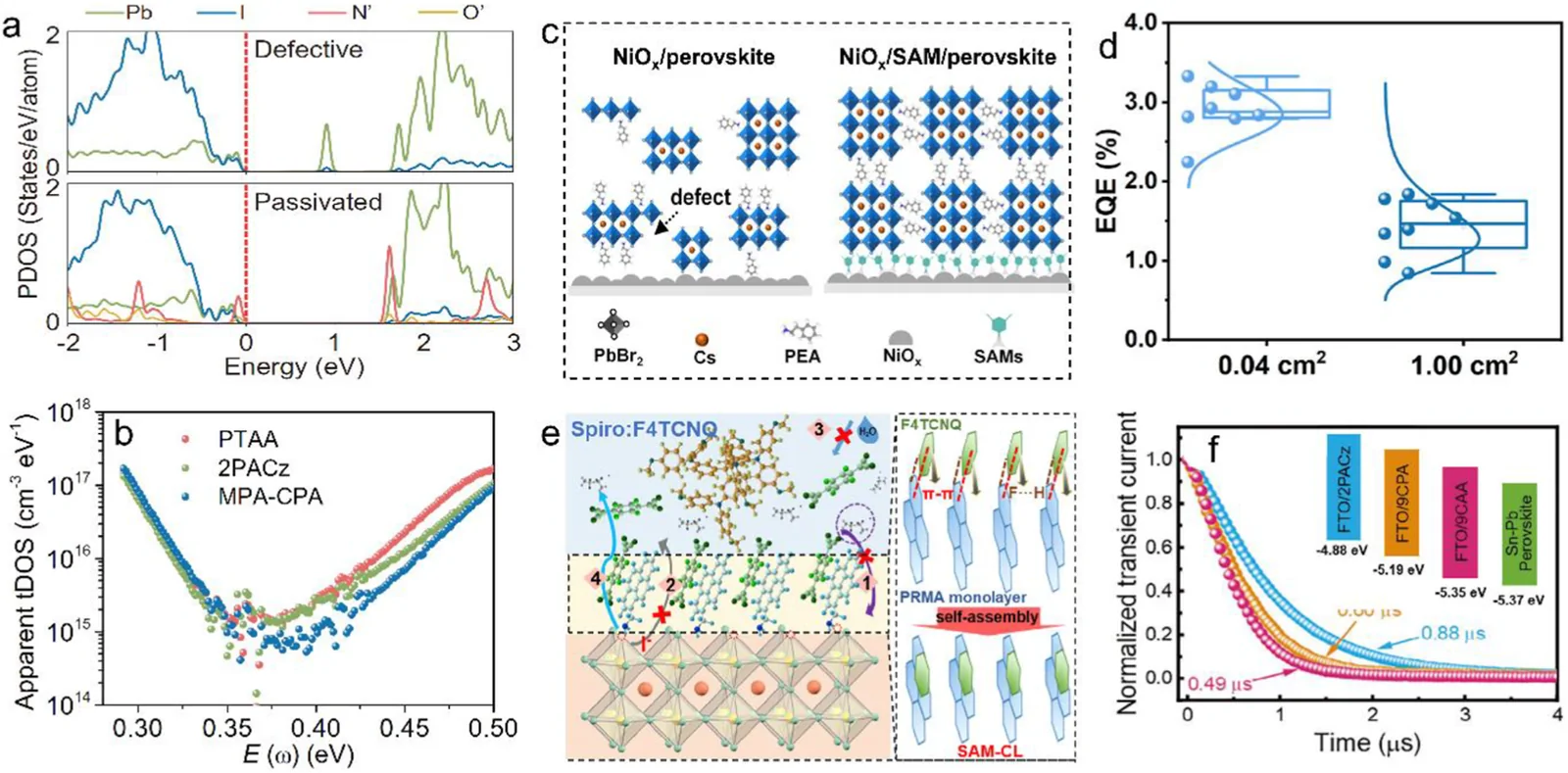
The photoelectronic properties of devices. **(a)** The density of states after optimized with SAMs. **(b)** The apparent trap density of states obtained by thermal admittance spectroscopy for devices based on different HTLs (Zhang S. et al., 2023). **(c)** Schematic illustration of the crystallization process of air-processed perovskites modified by SAMs. **(d)** The statistics of EQE of the PeLEDs on the Br-2PACz-modified substrate (Wu J. et al., 2026). **(e)** Schematic illustration of the assembly process and interfacial operating mechanism about SAM-CL (Wang X. et al., 2024). **(f)** The statistics of hole extraction and transport at the buried interface after treated with SAMs (Zhang Z. et al., 2024).

Recently, self-assembled monolayers (SAMs) have emerged as a prominent strategy for regulating charge injection and passivating buried interface defects in PeLEDs. The SAM optimizing photoelectronic performance of devices through the following four mechanisms: regulating carrier transport and energy level of transport layer, Improving the wetting property and uniformity of the film, releasing interface stress, and enhancing chemical stability of functional layer (Zhang S. et al., 2023; Chen et al., 2025; Wang X. et al., 2025). Firstly, SAMs anchored at the perovskite grain interface effectively passivated the typical deep-level defects such as interstitial lead (Pb_i_) and lead-iodide antisite (Pb_I_). The introduced MPA-CPA molecule formed new chemical bonds, which passivated the trap states and moved the state-density contribution to band edges (Figure 6a) (Zhang S. et al., 2023). The device based on MPA-CPA exhibited a lower apparent trap density of states at around 0.4 eV, indicating the reduction of electronic defects and elimination of ion charges in perovskite (Figure 6b), which revealed the outstanding advantages of SAMs in optimizing performance of PeLEDs. Simultaneously, the deposited SAM enhanced the wetting between transport layer and perovskite film, which significantly improved the morphology of EML. Figure 6c schematically illustrated the optimization of perovskite crystalline quality mediated by the SAM (Wu J. et al., 2026). Owing to its strong pre-nucleation interaction with the perovskite precursor, Br-2PACz provided dense nucleation centers at the buried interface to guide the crystallization of a uniform, pinhole-free perovskite layer, thereby suppressing severe leakage current and boosting radiative exciton recombination (Zuo et al., 2015). SAM-modified champion devices demonstrated a nearly threefold improvement compared to control devices, alongside markedly enhanced spectral stability under continuous electrical bias (Figure 6d). Chen et al. proposed an innovative interface engineering, self-assembled cocrystal layer, to eliminate the interfacial charge accumulation. As shown in the schematic diagram of Figure 6e (Wang X. et al., 2024), the hydrophobic functional groups of SAM layer and their strong bonding with perovskite interface constructed a robust physical and chemical barrier at the interface, which effectively suppressed electric-field-induced ion migration and hindered oxygen/moisture invasion, ultimately safeguarding the long-term electroluminescent operational stability of PeLEDs. Moreover, SAMs also tuned the substrate work function through oriented molecular dipoles and tailored structural modifications, establishing optimal interfacial energy-level alignment with the perovskite EML. As demonstrated in Figure 6f (Zhang Z. et al., 2024), the carrier injection barrier was significantly reduced, the interfacial carrier injection kinetics were accelerated and balanced, which was attributed to an efficient radiative recombination and lowered turn-on voltages.

### 4.2 Device architecture, thermal management, and outcoupling engineering

Beyond chemical engineering, physical device-architecture optimization plays a pivotal role in realizing high-performance PeLEDs. Previous literature indicates that approximately 70%–85% of internally generated photons remain trapped within the devices, hence overcoming the severe photonic waveguiding constraints and electro-thermal degradation is essential for enhancing optoelectronic performance (Marcato et al., 2024). Specifically, employing anisotropic, low-refractive-index and high-electron-mobility charge transport layers has proven effective in mitigating surface plasmon polariton losses and improving the light outcoupling efficiency of PeLEDs (Sun et al., 2024). Furthermore, 3D-printed micro-lens arrays on the outer substrate surface further improved the light extraction efficiency (Yue et al., 2025). Concurrently, monolithic tandem architectures that vertically integrate multiple perovskite emitting units lower the driving current density per unit, suppressing destructive Joule heating and extending operational durability (Wang Z. et al., 2025). Crucially, this stacked configuration leveraged interlayer photon recycling to re-extract photons trapped in internal waveguide modes, substantially boosting light extraction efficiency and driving the device to a record-high peak EQE exceeding 45.5% (Ke et al., 2025).

By harmonizing device semiconductor physics, interfacial coordination chemistry, thermal management, and optical wave engineering, these holistic device-level strategies enable simultaneous enhancements in efficiency, operational durability, and closed-loop lifecycle lead sequestration. Ultimately, these multi-dimensional advancements establish a robust foundation for the sustainable commercialization of PeLEDs in next-generation display and solid-state lighting technologies.

## 5 Conclusion and outlook

PeLEDs have been established as prominent contenders for next-generation display and lighting technologies. The [PbX_6_]^4-^ octahedral framework endowed perovskite outstanding optoelectronic properties including high PLQY, narrow FWHM, intrinsic defect tolerance, and remarkable electric performance, indicating conspicuous prospects in industrial photoelectric devices. However, the thermodynamic fragility, field-assisted ion migration, structural degradation, and potential toxicity concerns of Pb, still hindered the continuous applications.

To resolve this central contradiction, holistic advances have been demonstrated across the material-to-device spectrum. (1) Lattice regulation and Surface engineering: Compositional substitution and cation doping contracted the octahedral framework and elevated vacancy formation energies, thereby locking the lead-halide lattice. Concurrently, advanced multidentate ligands, core-shell architectures, and crystalline matrix encapsulations established physical and chemical barriers against environmental stress and lead leakage. (2) Eco-friendly synthesis and lead-free pursuing: Green, solvent-free mechanochemical paradigms and raw-material-selection strategies bypassed hazardous organic solvents, paving low-carbon, zero-waste pathways for perovskite processing. The lead-free perovskite provided another pathway to address the Pb-concerns in devices. (3) Interfacial optimization and device manufacturing: Transport-layer engineering and SAMs effectively eliminated buried interfacial traps, aligned energy levels, and suppressed charge accumulation. In tandem with photonic waveguiding management and monolithic stacked configurations, significant improvements in EQE and operating stability have been achieved in PeLEDs, alongside suppressed operational Joule heating and capability deterioration.

Despite these remarkable achievements, the practical deployment and commercial viability of PeLEDs necessitate further exploration across several key frontiers: (1) Atomic-level mechanism characterization. Developing advanced atomic-level characterization strategies enables a fundamental understanding of the intrinsic mechanisms driving perovskite operational failure, ion migration, and environment-mediated degradation. Uncovering these microscopic degradation pathways provides essential guidelines to fundamentally design targeted suppression methods and tailored passivation strategies for long-term device durability. (2) Ligand-mediated surface reconstruction. The surface-state defects remain the primary thermodynamic triggers for ion migration, phase degradation and nonradiative-recombination traps. Implementing ligand-mediated surface reconstruction strategies offers a compelling pathway to reconfigure surface coordination states and enhance the structural physical/chemical stability, thereby guaranteeing long-term resilience and operational durability of perovskite emissive layers. (3) Balanced carrier injection. Further developing novel dual-functional conductive SAMs, can concurrently anchor undercoordinated Pb^2+^ defects via electron-donating coordination and facilitate low-barrier, balanced carrier injection, thereby effectively fabricating high-performance and long operating-lifetime devices. (4) End-to-end manufacturing and management for commercialization. Integrating automated solvent-free synthesis with scalable printing establishes end-to-end manufacturing workflows essential for large-area commercial display production. Simultaneously, deploying process-wide management protocols that encompass lead-sequestration encapsulation and eco-friendly recycling, guarantees strict environmental compliance across the entire product lifecycle.

Predictably, a holistic synergy combining fundamental material synthesis, interfacial device physics, and closed-loop lifecycle lead management will pave a sustainable pathway toward the high-performance, long-term stability and scalable commercialization of next-generation PeLED for display and lighting technologies.

## References

[B1] Ahmed G. H. Yin J. Bakr O. M. Mohammed O. F. (2021). Successes and challenges of core/shell lead halide perovskite nanocrystals. ACS Energy Lett. 6 (4), 1340–1357. 10.1021/acsenergylett.1c00076

[B2] Aristidou N. Sanchez-Molina I. Chotchuangchutchaval T. Brown M. Martinez L. Rath T. (2015). The role of oxygen in the degradation of methylammonium lead trihalide perovskite photoactive layers. Angew. Chem. Int. Ed. 54 (28), 8208–8212. 10.1002/anie.201503153 26014846

[B3] Bai S. Yuan Z. Gao F. (2016). Colloidal metal halide perovskite nanocrystals: synthesis, characterization, and applications. J. Mater. Chem. C 4 (18), 3898–3904. 10.1039/c5tc04116c

[B4] Bai W. Xuan T. Zhao H. Dong H. Cheng X. Wang L. (2023). Perovskite light-emitting diodes with an external quantum efficiency exceeding 30%. Adv. Mater. 35 (39), e2302283. 10.1002/adma.202302283 37246938

[B5] Birkhold S. T. Precht J. T. Liu H. Giridharagopal R. Eperon G. E. Schmidt-Mende L. (2018). Interplay of Mobile ions and injected carriers creates recombination centers in metal halide perovskites under bias. ACS Energy Lett. 3 (6), 1279–1286. 10.1021/acsenergylett.8b00505

[B6] Brandt R. E. Stevanović V. Ginley D. S. Buonassisi T. (2015). Identifying defect-tolerant semiconductors with high minority-carrier lifetimes: beyond hybrid lead halide perovskites. MRS Commun. 5 (2), 265–275. 10.1557/mrc.2015.26

[B7] Cai J. Zhang X. Chen Y. Lai W. Ye Y. Xu S. (2025). Neuron-inspired CsPbBr_3_/PDMS nanospheres for multi-dimensional sensing and interactive displays. Light Sci. and Appl. 14 (1), 55. 10.1038/s41377-025-01742-z PMC11742394 39824818

[B8] Chen Z. Li Z. Hopper T. R. Bakulin A. A. Yip H.-L. (2021). Materials, photophysics and device engineering of perovskite light-emitting diodes. Rep. Prog. Phys. 84 (4), 046401. 10.1088/1361-6633/abefba 33730709

[B9] Chen P. Hou J. Wang L. (2022). Metal-organic framework-tailored perovskite solar cells. Microstructures 2 (3), 14. 10.20517/microstructures.2022.05

[B10] Chen B. He X. Yu J. He Z. Peng C. Zhang F. (2025). Hybrid self-assembled molecules reduce interfacial defects in perovskite light-emitting diodes. ACS Energy Lett. 10 (8), 4013–4018. 10.1021/acsenergylett.5c01884

[B11] Chu Y. Zhang W. Hu B. Wen J. Qin M. Zhao G. (2025). Unveiling the surface exciton dynamics in K-Doped perovskite quantum dots using chiral ligands enables efficient white LEDs. ACS Sustain. Chem. and Eng. 13 (31), 12680–12690. 10.1021/acssuschemeng.5c04871

[B12] Do T. H. Loganathan A. Yin Y. F. Lin X. Y. Fu Y. S. Guo T. F. (2025). Clarify the cathode degradation in perovskite-based light-emitting diodes. Adv. Funct. Mater. 36 (14), e21392. 10.1002/adfm.202521392

[B13] Dong Q. Lei L. Mendes J. So F. (2020). Operational stability of perovskite light emitting diodes. J. Phys. Mater. 3 (1), 012002. 10.1088/2515-7639/ab60c4

[B14] Fabini D. H. Seshadri R. Kanatzidis M. G. (2020). The underappreciated lone pair in halide perovskites underpins their unusual properties. MRS Bull. 45 (6), 467–477. 10.1557/mrs.2020.142

[B15] Fan W. Wang S. Gong C. Feng Z. Li B. Yang Z. (2025a). Synthesis of highly efficient and dispersible OM-CsPbBr3 quantum dots integrated into polymers by surface medium anchoring strategy. Chem. Eng. J. 520, 165951. 10.1016/j.cej.2025.165951

[B16] Fan W. Wang S. Yang Z. Yao J. Xu L. Song J. (2025b). *In situ* formation of luminescent perovskite quantum dot/polymer composites: scalable synthesis, continuous processing and functional applications. Adv. Mater. 37 (39), e2505600. 10.1002/adma.202505600 40629996

[B17] Fiuza-Maneiro N. Sun K. López-Fernández I. Gómez-Graña S. Müller-Buschbaum P. Polavarapu L. (2023). Ligand chemistry of inorganic lead halide perovskite nanocrystals. ACS Energy Lett. 8 (2), 1152–1191. 10.1021/acsenergylett.2c02363

[B18] Futscher M. H. Gangishetty M. K. Congreve D. N. Ehrler B. (2020). Manganese doping stabilizes perovskite light-emitting diodes by reducing ion migration. ACS Appl. Electron. Mater. 2 (6), 1522–1528. 10.1021/acsaelm.0c00125

[B19] Gan Y. Shi Y. Ghosh S. Liu H. Xu H. Xiong Q. (2025). Ultrafast neuromorphic computing driven by polariton nonlinearities. eLight 5 (1), 9. 10.1186/s43593-025-00087-9

[B20] Gao Y. Cai Q. He Y. Zhang D. Cao Q. Zhu M. (2024). Highly efficient blue light-emitting diodes based onmixed-halide perovskites with reduced chlorine defects. Sci. Adv. 10 (29), eado5645. 10.1126/sciadv.ado5645 39018409 PMC466955

[B21] Goyal A. Andrioti E. Tang Y. Zhao Q. Zheng K. Newell K. D. (2022). Mechanochemical synthesis of stable, quantum-confined CsPbBr_3_ perovskite nanocrystals with blue-green emission and high PLQY. J. Phys. Mater. 5 (2), 024005. 10.1088/2515-7639/ac618f

[B22] Guo K. Li N. Zhang M. Ren L. Qiao F. Pan S. (2025). Efficient all-solution-processed perovskite light-emitting diodes via a room-temperature vapor-treated interlayer. ACS Appl. Mater. and Interfaces 17 (10), 15688–15697. 10.1021/acsami.4c19098 39999340

[B23] Han T. H. Jang K. Y. Dong Y. T. Friend R. H. Sargent E. H. Lee T. W. (2022). A roadmap for the commercialization of perovskite light emitters. Nat. Rev. Mater. 7 (10), 757–777. 10.1038/s41578-022-00459-4

[B24] Han D. Wang J. Agosta L. Zang Z. Zhao B. Kong L. (2023). Tautomeric mixture coordination enables efficient lead-free perovskite LEDs. Nature 622 (7983), 493–498. 10.1038/s41586-023-06514-6 37557914

[B25] Hu B. Y. Zhang W. Q. Zhao G. J. Rosei F. Zhang X. X. Zhao J. S. (2025). Unveiling self-trapped exciton emission in lanthanide-doped Cs_2_NaInCl_6_ double perovskites for efficient white LEDs. Rare Met. 44 (11), 8849–8862. 10.1007/s12598-025-03475-9

[B26] Jang E. Jang H. (2023). Review: quantum dot light-emitting diodes. Chem. Rev. 123 (8), 4663–4692. 10.1021/acs.chemrev.2c00695 36795794

[B27] Jiang J. Xia Z. Shi M. Yin Z. Huang W. Zeng L. (2025). Efficient red perovskite LEDs with iodine management via volatile additive I2. Adv. Mater. 37 (32), 2503699. 10.1002/adma.202503699 40405631

[B28] Karlsson M. Yi Z. Reichert S. Luo X. Lin W. Zhang Z. (2021). Mixed halide perovskites for spectrally stable and high-efficiency blue light-emitting diodes. Nat. Commun. 21, 361–370. 10.1038/s41467-020-20582-6 33441549 PMC7806600

[B29] Ke Y. Zhu W. Ma C. Xiong K. Liu W. Kuang Z. (2025). High-performance tandem perovskite LEDs through interlayer photon recycling. Nature 649 (8095), 53–58. 10.1038/s41586-025-09865-4 41219499

[B30] Kim J. S. Heo J.-M. Park G.-S. Woo S.-J. Cho C. Yun H. J. (2022). Ultra-bright, efficient and stable perovskite light-emitting diodes. Nature 611 (7937), 688–694. 10.1038/s41586-022-05304-w 36352223

[B31] Kong L. Zhang X. Zhang C. Wang L. Wang S. Cao F. (2022). Stability of perovskite light-emitting diodes: existing issues and mitigation strategies related to both material and device aspects. Adv. Mater. 34 (43), e2205217. 10.1002/adma.202205217 35921550

[B32] Kovalenko M. Bovgyra O. Chornodolskyy Y. Pidhornyi O. Pushak A. Voloshinovskii A. (2025). Electronic structure of sr-doped CsPbCl_3_ crystal: first-principles investigation. Low. Temp. Phys. 51 (3), 339–347. 10.1063/10.0035837

[B33] Kundu S. Kelly T. L. (2020). *In situ* studies of the degradation mechanisms of perovskite solar cells. EcoMat 2 (2), e12025. 10.1002/eom2.12025

[B34] Li J. H. Xu L. M. Wang T. Song J. Z. Chen J. W. Xue J. (2017). 50-Fold EQE improvement up to 6.27% of solution-processed all-inorganic perovskite CsPbBr3 QLEDs via surface ligand density control. Adv. Mater. 29 (5), 9. 10.1002/adma.201603885 27882606

[B35] Li J. Chen J. Xu L. Liu S. Lan S. Li X. (2020). A zinc non-halide dopant strategy enables efficient perovskite CsPbI3 quantum dot-based light-emitting diodes. Mater. Chem. Front. 4 (5), 1444–1453. 10.1039/c9qm00734b

[B36] Li N. Jia Y. Guo Y. Zhao N. (2022). Ion migration in perovskite light-emitting diodes: mechanism, characterizations, and material and device engineering. Adv. Mater. 34 (19), e2108102. 10.1002/adma.202108102 34847262

[B37] Li Y. Guan X. Zhao Y. Zhang Q. Chen X. Zhang S. (2024). Modulation of charge transport layer for perovskite light-emitting diodes. Adv. Mater. 37 (25), e2410535. 10.1002/adma.202410535 39443833

[B38] Li X. Gu Q. Huang W. Fairclough S. M. Friend R. H. Stranks S. D. (2026). Multimodal electron microscopy of halide perovskite interfacial dynamics. Nature 651 (8106), 614–620. 10.1038/s41586-026-10238-8 41813888 PMC12999523

[B39] Liang Y. Li F. Cui X. Stampfl C. Ringer S. P. Yang X. (2025). Multiple B-site doping suppresses ion migration in halide perovskites. Sci. Adv. 11, eads7054. 10.1126/sciadv.ads7054 40085694 PMC11908468

[B40] Lin C. C. Wan S. C. Shen C. H. Liao Z. L. Liu Y. Wu Z. Y. (2025). Achieving long-term water stability and strong exciton–photon coupling in CsPbBr_3_ quantum dots via MOF encapsulation. Nanophotonics 14 (14), 2397–2409. 10.1515/nanoph-2025-0059 40687568 PMC12273544

[B41] Liu W. Qiao Y. Deng Y. Zhang Y. Liu C. Guo W. (2025). Advancements and challenges of highly efficient and stable perovskite light-emitting diodes. Laser and Photonics Rev. 20 (2), e01787. 10.1002/lpor.202501787

[B42] Liu N. Li Z. Xiang Z. Luo J. Chen C. Tang J. (2026). Focus on performance of perovskite light-emitting diodes (2026). Front. Optoelectron. 19 (2), 14. 10.2738/foe.2026.0014 PMC9743889 36641572

[B43] Maietta I. Otero-Martínez C. Fernández S. Sánchez L. González-Fernández Á. Polavarapu L. (2025). The toxicity of lead and lead-free perovskite precursors and nanocrystals to human cells and aquatic organisms. Adv. Sci. 12 (13), e2415574. 10.1002/advs.202415574 PMC11967815 39927780

[B44] Marcato T. Kumar S. Shih C. J. (2024). Strategies for controlling emission anisotropy in lead halide perovskite emitters for LED outcoupling enhancement. Adv. Mater. 37 (25), e2413622. 10.1002/adma.202413622 39676496 PMC12204137

[B45] Mirabelli A. J. Kammlander B. Lu Y. Varma R. M. Gu Q. Radetzky K. (2025). Interfacial chemistry limits the stability of deep blue perovskite LEDs revealed by operando characterization. ACS Energy Lett. 10 (7), 3533–3543. 10.1021/acsenergylett.5c00989 40672129 PMC12261314

[B46] Mosquera-Lois I. Huang Y.-T. Lohan H. Ye J. Walsh A. Hoye R. L. Z. (2025). Multifaceted nature of defect tolerance in halide perovskites and emerging semiconductors. Nat. Rev. Chem. 9 (5), 287–304. 10.1038/s41570-025-00702-w 40195529

[B47] Patel M. Patel R. Park C. Cho K. Kumar P. Park C. (2023). Water-stable, biocompatible, and highly luminescent perovskite nanocrystals-embedded fiber-based paper for anti-counterfeiting applications. Nano Converg. 10 (1), 21. 10.1186/s40580-023-00366-6 37133613 PMC10156878

[B48] Peng X. F. Wu X. Y. Ji X. X. Ren J. Wang Q. Li G. Q. (2017). Modified conducting polymer hole injection layer for high-efficiency perovskite light-emitting devices: enhanced hole injection and reduced luminescence quenching. J. Phys. Chem. Lett. 8 (19), 4691–4697. 10.1021/acs.jpclett.7b01992 28914543

[B49] Peng J. Xue X. Liu S. Yang Y. Yang T. Zhu B. (2026). Maximizing perovskite electroluminescence with ordered 3D/2D heterojunction. Nature 651 (8104), 76–82. 10.1038/s41586-026-10134-1 41673160

[B50] Protesescu L. Yakunin S. Nazarenko O. Dirin D. N. Kovalenko M. V. (2018). Low-cost synthesis of highly luminescent colloidal lead halide perovskite nanocrystals by wet ball milling. ACS Appl. Nano Mater. 1 (3), 1300–1308. 10.1021/acsanm.8b00038 29911683 PMC5999230

[B51] Ravi V. K. Mondal B. Nawale V. V. Nag A. (2020). Don’t let the lead out: new material chemistry approaches for sustainable lead halide perovskite solar cells. ASCLungNano Lett. Omega 5 (46), 29631–29641. 10.1021/acsomega.0c04599 33251399 PMC7689680

[B52] Shan Q. Wei C. Jiang Y. Song J. Zou Y. Xu L. (2020). Perovskite light-emitting/detecting bifunctional fibres for wearable LiFi communication. Light Sci. and Appl. 9 (1), 163. 10.1038/s41377-020-00402-8 33014358 PMC7494868

[B53] Shen X. Zhang Y. Kershaw S. V. Li T. Wang C. Zhang X. (2019). Zn-Alloyed CsPbI_3_ nanocrystals for highly efficient perovskite light-emitting devices. Nano Lett. 19 (3), 1552–1559. 10.1021/acs.nanolett.8b04339 30741555

[B54] Shi Y. Chang S. E. Lee T.-W. (2025). Sustainable perovskite light emitters. Chem. Soc. Rev. 54 (22), 10316–10325. 10.1039/d5cs00620a 41031460

[B55] Song J. Z. Li J. H. Xu L. M. Li J. H. Zhang F. J. Han B. N. (2018). Room-temperature triple-ligand surface engineering synergistically boosts ink stability, recombination dynamics, and charge injection toward EQE-11.6% perovskite QLEDs. Adv. Mater. 30 (30), 7. 10.1002/adma.201800764 29888521

[B56] Stranks S. D. Snaith H. J. (2015). Metal-halide perovskites for photovoltaic and light-emitting devices. Nat. Nanotechnol. 10 (5), 391–402. 10.1038/nnano.2015.90 25947963

[B57] Sun S. Yuan D. Xu Y. Wang A. Deng Z. (2016). Ligand-mediated synthesis of shape-controlled cesium lead halide perovskite nanocrystals via reprecipitation process at room temperature. ACS Nano 10 (3), 3648–3657. 10.1021/acsnano.5b08193 26886173

[B58] Sun Y. Ge L. Dai L. Cho C. Ferrer Orri J. Ji K. (2023). Bright and stable perovskite light-emitting diodes in the near-infrared range. Nature 615 (7954), 830–835. 10.1038/s41586-023-05792-4 36922588

[B59] Sun S. Q. Tai J. W. He W. Yu Y. J. Feng Z. Q. Sun Q. (2024). Enhancing light outcoupling efficiency via anisotropic low refractive index electron transporting materials for efficient perovskite light-emitting diodes. Adv. Mater. 36 (24), e2400421. 10.1002/adma.202400421 38430204

[B60] Sun S.-Q. Sun Q. Cai Y. Feng Z.-Q. Zheng Q. Liu B. (2025). Mitigation of nonradiative recombination by reconfiguring triplet energy of additive toward efficient blue perovskite light-emitting diodes. ACS Nano 19 (13), 13053–13062. 10.1021/acsnano.4c18116 40136061

[B61] Tang W. D. Liu S. N. Zhang G. Ren Z. X. Liu Z. Zhang M. (2025). Lead-free perovskite light-emitting diodes. Adv. Mater. 37 (25), e2411020. 10.1002/adma.202411020 39449210

[B62] Tao S. Schmidt I. Brocks G. Jiang J. Tranca I. Meerholz K. (2019). Absolute energy level positions in tin- and lead-based halide perovskites. Nat. Commun. 10 (1), 2560. 10.1038/s41467-019-10468-7 31189871 PMC6561953

[B63] Tatarinov D. A. Ismagilov A. O. Koroleva A. V. Zhizhin E. V. Zheng W. Baranov A. V. (2025). Enhanced stability and optical performance of CsPbBr_3_@FAPbBr_3_ core–shell perovskite nanocrystals. Nanoscale 17 (11), 6695–6703. 10.1039/d4nr05049e 39957249

[B64] Thiesbrummel J. Milić J. V. Deibel C. Garnett E. C. Tao S. Kirchartz T. (2026). Ion migration in perovskite solar cells. Nat. Rev. Chem. 10 (3), 179–195. 10.1038/s41570-025-00790-8 41566017

[B65] Tsai H. Nie W. Blancon J.-C. Stoumpos C. C. Asadpour R. Harutyunyan B. (2016). High-efficiency two-dimensional Ruddlesden–Popper perovskite solar cells. Nature 536 (7616), 312–316. 10.1038/nature18306 27383783

[B66] Umebayashi T. Asai K. Kondo T. Nakao A. (2003). Electronic structures of lead iodide based low-dimensional crystals. Phys. Rev. B 67 (15), 155405. 10.1103/PhysRevB.67.155405

[B67] Vashishtha P. Halpert J. E. (2017). Field-driven ion migration and color instability in red-emitting mixed halide perovskite nanocrystal light-emitting diodes. Chem. Mater. 29 (14), 5965–5973. 10.1021/acs.chemmater.7b01609

[B68] Wang X. Zhong Y. Luo X. Sheng W. Yang J. Tan L. (2024a). Elimination of charge accumulation by a self-assembled cocrystal interlayer for efficient and stable perovskite solar cells. Energy and Environ. Sci. 17 (2), 569–579. 10.1039/d3ee03550f

[B69] Wang Y.-K. Wan H. Teale S. Grater L. Zhao F. Zhang Z. (2024b). Long-range order enabled stability in quantum dot light-emitting diodes. Nature 629 (8012), 586–591. 10.1038/s41586-024-07363-7 38720080

[B70] Wang X. Wang X. Wang X. Li M. Li H. Fu Y. (2025a). Role of self-assembled molecules in halide perovskite optoelectronics: an atomic-scale perspective. Natl. Sci. Rev. 12 (5), nwaf150. 10.1093/nsr/nwaf150 PMC12096312 40406599

[B71] Wang Z. Zhang G. Ren Z. Yang Y. Wang Z. Chen H. (2025b). Enhancing thermal tolerance for bright and stable near-infrared perovskite LEDs. Adv. Mater. 37 (32), e2502659. 10.1002/adma.202502659 40405624

[B72] Wei K. Zhou T. Jiang Y. Sun C. Liu Y. Li S. (2025). Perovskite heteroepitaxy for high-efficiency and stable pure-red LEDs. Nature 638 (8052), 949–956. 10.1038/s41586-024-08503-9 39972133

[B73] Woo S.-J. Kim J. S. Lee T.-W. (2021). Characterization of stability and challenges to improve lifetime in perovskite LEDs. Nat. Photonics 15 (9), 630–634. 10.1038/s41566-021-00863-2

[B74] Wu J. Wang Y. Lin F. Ni C. Xiong S. Zhang M. (2026a). Self-assembled monolayer modified nickel oxide surface for air-processed blue perovskite light-emitting diodes. RSC Appl. Interfaces 3 (3), 687–695. 10.1039/d5lf00384a

[B75] Wu R. Gong S. Wu Y. Ren Z. Marongiu D. Saba M. (2026b). Core–shell engineering of CsPbBr_3_ nanocrystals: structures, properties, and applications. Physica Status Solidi (RRL) – Rapid Res. Lett. 20 (3), e202500439. 10.1002/pssr.202500439

[B76] Wu X. T. Hu T. J. Shan Q. S. Zhong H. Su Y. Q. Yang L. X. (2026c). Ligand-mediated intergrain isolation strategy for narrow-emission deep-blue perovskite quantum dot light-emitting diodes. Acs Photonics 13 (5), 1537–1545. 10.1021/acsphotonics.6c00072

[B77] Xie M. Guo J. Zhang X. Bi C. Zhang L. Chu Z. (2022). High-efficiency pure-red perovskite quantum-dot light-emitting diodes. Nano Lett. 22 (20), 8266–8273. 10.1021/acs.nanolett.2c03062 36251485

[B78] Yang D. Li X. Zhou W. Zhang S. Meng C. Wu Y. (2019). CsPbBr_3_ quantum dots 2.0: benzenesulfonic acid equivalent ligand awakens complete purification. Adv. Mater. 31 (30), e1900767. 10.1002/adma.201900767 31172615

[B79] Yang D. Zhang G. Lai R. Cheng Y. Lian Y. Rao M. (2021). Germanium-lead perovskite light-emitting diodes. Nat. Commun. 12 (1), 4295. 10.1038/s41467-021-24616-5 34257298 PMC8277869

[B80] Yin W.-J. Shi T. Yan Y. (2014). Unusual defect physics in CH3NH3PbI3 perovskite solar cell absorber. Appl. Phys. Lett. 104, 063903. 10.1063/1.4864778

[B81] You J. Y. Lv R. Q. Zhang F. Y. Wang B. Chen J. Zeng H. B. (2026). Laser-induced *in situ* synthesis of perovskite quantum dots: mechanisms and patterned applications. Adv. Opt. Mater. 14 (6), 26. 10.1002/adom.202503664

[B82] Yuan Z. Miao Y. Hu Z. Xu W. Kuang C. Pan K. (2019). Unveiling the synergistic effect of precursor stoichiometry and interfacial reactions for perovskite light-emitting diodes. Nat. Commun. 10 (1), 2818. 10.1038/s41467-019-10612-3 31249295 PMC6597563

[B83] Yuan S. Zheng X. Shen W.-S. Liu J. Cui L.-S. Zhang C. (2022). Overcoming degradation pathways to achieve stable blue perovskite light-emitting diodes. ACS Energy Lett. 7 (4), 1348–1354. 10.1021/acsenergylett.2c00465

[B84] Yuan Z. Bai S. Gao F. Snaith H. J. (2025). Influence of interfacial reactions on perovskite optoelectronic devices. Small Methods 9 (10), e2500438. 10.1002/smtd.202500438 40317672 PMC12536404

[B85] Yue Y. Chen K. Zhu H. Zhu P. (2025). Advancing LED efficiency through 3D-printed light extraction structures: from design to demonstration. Opt. Express 33 (21), 44434–44453. 10.1364/oe.572911 41215230

[B86] Zhang C. Wang S. Li X. Yuan M. Turyanska L. Yang X. (2020). Core/shell perovskite nanocrystals: synthesis of highly efficient and environmentally stable FAPbBr_3_/CsPbBr_3_ for LED applications. Adv. Funct. Mater. 30 (31), 1910582. 10.1002/adfm.201910582

[B87] Zhang S. Ye F. Wang X. Chen R. Zhang H. Zhan L. (2023a). Minimizing buried interfacial defects for efficient inverted perovskite solar cells. Science 380, 404–409. 10.1126/science.adg3755 37104579

[B88] Zhang L. X. Mei L. Y. Wang K. Y. Lv Y. H. Zhang S. Lian Y. X. (2023b). Advances in the application of perovskite materials. Nano-Micro Lett. 15 (1), 177. 10.1007/s40820-023-01140-3 PMC10333173 37428261

[B89] Zhang X. Cai J. Yang L. Luo J. Guo T. Chen E. (2024a). Ligand strategy for perovskite displays: a review. ACS Energy Lett. 9 (4), 1587–1603. 10.1021/acsenergylett.3c02454

[B90] Zhang Z. Zhu R. Tang Y. Su Z. Hu S. Zhang X. (2024b). Anchoring charge selective self-assembled monolayers for tin-lead perovskite solar cells. Adv. Mater. 36 (18), e2312264. 10.1002/adma.202312264 38281081

[B91] Zhang M. Ma X. Esguerra J. L. Yu H. Hjelm O. Li J. (2025a). Towards sustainable perovskite light-emitting diodes. Nat. Sustain. 8 (3), 315–324. 10.1038/s41893-024-01503-7

[B92] Zhang X. Huang H. Zhao C. Yuan J. (2025b). Surface chemistry-engineered perovskite quantum dot photovoltaics. Chem. Soc. Rev. 54 (6), 3017–3060. 10.1039/d4cs01107d 39962988

[B93] Zhang W. Chu Ya Hu B. Xu L. Zhao G. Sund Y. (2026). Lead-free perovskites for photocatalysis: Synthesis, mechanisms and applications. Chin. Chem. Lett. 112747. 10.1016/j.cclet.2026.112747

[B94] Zheng D. G. Kim D. H. (2022). Degradation mechanisms of perovskite light-emitting diodes under electrical bias. Nanophotonics 12 (3), 451–476. 10.1515/nanoph-2022-0569 39635398 PMC11502103

[B95] Zuo L. Gu Z. Ye T. Fu W. Wu G. Li H. (2015). Enhanced photovoltaic performance of CH3NH3PbI3 perovskite solar cells through interfacial engineering using self-assembling monolayer. J. Am. Chem. Soc. 137 (7), 2674–2679. 10.1021/ja512518r 25650811

